# Are Artemisinin-Based Combination Therapies For Malaria Beginning To Fail in Africa?

**DOI:** 10.4269/ajtmh.21-0797

**Published:** 2021-09-07

**Authors:** Philip J. Rosenthal

**Affiliations:** Department of Medicine, University of California, San Francisco, California

Despite advances in many countries, notably the recent declaration that China is now malaria free, malaria remains entrenched in Africa, where over 90% of malaria morbidity and mortality is seen.^1^ In the past, Africa suffered greatly from the effects of resistance to chloroquine, the mainstay of malaria treatment for decades, with increasing mortality from *Plasmodium falciparum* over the last decades of the 20th century. These increases were turned around early this century, with an estimated 57% decrease in the rate of malaria deaths from 2000 to 2015.^2^ A major factor contributing to this decrease was availability of highly effective artemisinin-based combination therapies (ACTs) to treat malaria.

Although ACTs helped to stem the rising tide of malaria mortality in Africa, they faltered in Asia, with delayed clearance of parasites after treatment with artemisinins, generally referred to as artemisinin resistance (or partial resistance), first identified in Cambodia more than a decade ago. Important studies have documented the spread of artemisinin resistance through the Greater Mekong subregion,^3^ the association of resistance with mutations in the *P. falciparum kelch13* (K13) gene,^4^ and loss of treatment efficacy of some ACTs, notably artesunate-mefloquine^5^ and dihydroartemisinin-piperaquine,^6^ as emergence of resistance to partner drugs followed that to artemisinins.

Of great concern is the potential for spread of ACT resistance (failures due to resistance to both components of the combination) to Africa, with a repeat of what was seen with chloroquine resistance in the last century, with many excess deaths due to the inability of available therapies to effectively treat falciparum malaria. At present, most evidence suggests that multiple ACTs continue to offer excellent therapeutic efficacy against malaria in Africa.^7^^,^^8^ Yet, some recent reports suggest that we may be seeing early signs of ACT resistance, as will be discussed below.

More than 100 different mutations have been identified in the *P. falciparum* K13 gene, with 10 deemed validated markers of artemisinin resistance and another 11, with more limited available data, considered candidate or associated markers by the WHO.^8^ Recently, some of these mutations have been seen to emerge in Africa. The K13 561H mutation, a validated resistance marker, has been seen at up to ∼20% prevalence at sites in Rwanda, and it was associated with delayed parasite clearance (but not treatment failure of artemether-lumefantrine) in a clinical trial.^9^^,^^10^ Two other K13 mutations that are candidate (675V) or associated (469Y) resistance markers have been seen at prevalences over 10% at multiple sites in northern Uganda,^11^ and unpublished reports have described additional relevant mutations seen in other countries. The full clinical implications of these K13 mutations are as yet unclear, but recent trials in Rwanda^9^ and Uganda^12^ have shown excellent ACT treatment efficacy, perhaps due to continued excellent efficacy of artemisinin partner drugs. However, there is concern that Africa may see the pattern experienced in southeast Asia, with emergence of artemisinin resistance followed by decreased activity of partner drugs and then emergence of true ACT resistance. In fact, other data suggest that such resistance may already be emerging in Africa.

The WHO uses a cutoff of 90% to define acceptable malaria treatment efficacy. Use of a precise cutoff is somewhat problematic because measured efficacy may vary based on factors independent of drug resistance, including the immunity and genetics of local populations and details of trial design and data analysis, in particular varied molecular methods and analyses to distinguish recrudescences (true treatment failures) from new infections after therapy.^13^ Nonetheless, consideration of treatment efficacy over time is important.

Most studies of approved ACTs (artemether-lumefantrine, artesunate-amodiaquine, dihydroartemisinin-piperaquine, artesunate-mefloquine, and artesunate-pyronaridine) conducted in Africa in recent years following standard WHO protocols have shown excellent treatment efficacy.^7^ However, there have been important exceptions. A series of four studies conducted every 2 years in Angola from 2013 to 2019 showed treatment efficacies (values provided below were corrected by molecular methods to discriminate recrudescences from new infections, although analytical approaches varied) for artemether-lumefantrine below 90% for Zaire Province in 2013 and 2015 (but not 2017 or 2019) and for Lunda Sul Province in 2019; efficacies for the other tested ACTs (artesunate-amodiaquine and dihydroartemisinin-piperaquine) were mostly > 95%.^14^^–^^17^ A new report in this issue of the *American Journal of Tropical Medicine and Hygiene* describes the results of ACT treatment trials conducted at four sites in the Democratic Republic of the Congo (DRC) in 2017–2018.^18^ Most efficacies were > 90%, but in Mikalayi, which is near the border with Zaire Province, Angola, treatment efficacy was 86% for artemether-lumefantrine and 84% for dihydroartemisinin-piperaquine. Even more striking results were recently reported from Burkina Faso, with treatment efficacies of 74% and 76% for artemether-lumefantrine and 89% and 84% for dihydroartemisinin-piperaquine noted in two provinces.^19^ Importantly, the relatively low efficacy values in Angola and DRC were derived from a Bayesian algorithm for outcome assignment that can identify higher failure rates than the standard algorithm recommended by the WHO,^20^^,^^21^ and results from Burkina Faso are arguably difficult to interpret due to deviations from standard WHO protocols.^22^ Interestingly, for the reported studies from Angola, DRC, and Burkina Faso, sequencing did not identify K13 mutations associated with artemisinin resistance in southeast Asia.

Considering the results described above, are we beginning to see ACT resistance in Africa? The answer is not yet clear, but two independent trends deserve careful attention. First, K13 mutations previously associated with artemisinin resistance have emerged in multiple African countries, albeit without apparent loss of ACT treatment efficacy. Second, ACT treatment efficacies below 90% have been seen in some other African countries without apparent emergence of relevant K13 mutations. How should we act on these findings? For the present, with limited evidence for increasing rates of ACT treatment failure, continued use of artemether-lumefantrine and other approved ACTs to treat malaria in Africa is appropriate. However, frequent surveillance across Africa is needed to identify further emergence of genetic polymorphisms that may mediate resistance to artemisinins and their partner drugs, ex vivo evidence of decreased drug susceptibility, or failures of ACT treatment efficacy in well-conducted clinical trials. For the future, emergence of ACT resistance may necessitate new therapeutic approaches for malaria in Africa.
